# Supplementary material for: EnzML: multi-label prediction of enzyme classes using InterPro signatures
Source: BMC Bioinformatics. 2012 Apr 25;13:61. doi: 10.1186/1471-2105-13-61 (PMC3483700; doi:10.1186/1471-2105-13-61)
Supplement: Addtional file 5 — The Java code to format the data files, evaluate and predict. The file enzml_java_code.tar.gz contains the Java code used to format database data to ARFF and XML formats, to execute cross and train-test (jackknife) evaluations and to record evaluation results to database. More information is included in the readme.txt file and the Javadoc files. The code can be used with a MySQL database. To use a different database software, other JDBC drivers might be required. [file 1471-2105-13-61-S5.gz › java_code/enzml2011/doc/uk/ac/ed/inf/enzml/mulan/learn/traintest/TrainTestEvaluator.html]

TrainTestEvaluator


---


|  |  |  |
| --- | --- | --- |
| **Overview** \| **Package** \| **Class** \| **Use** \| **Tree** \| **Deprecated** \| **Index** \| **Help** | |  |
| **PREV CLASS**   **NEXT CLASS** | **FRAMES**    **NO FRAMES**     **All Classes** |  |
| SUMMARY: NESTED \| FIELD \| CONSTR \| METHOD | DETAIL: FIELD \| CONSTR \| METHOD |  |


---


## uk.ac.ed.inf.enzml.mulan.learn.traintest Class TrainTestEvaluator

```
java.lang.Object
  mulan.evaluation.Evaluator
      uk.ac.ed.inf.enzml.mulan.learn.EvaluatorNoAUC
          uk.ac.ed.inf.enzml.mulan.learn.MulanCrossEvaluator
              uk.ac.ed.inf.enzml.mulan.learn.traintest.TrainTestEvaluator
```

**Direct Known Subclasses:**: TrainTestEvaluatorSerialised

---

``` public class TrainTestEvaluator extends MulanCrossEvaluator ```

Train/test evaluator. The learner can be trained/built on the spot or can be
taken from a file containing an already trained/build serialised learner.

**Version:**
:   21 Oct 2010

**Author:**
:   Luna De Ferrari luna.deferrari-at-ed.ac.uk

---

**Constructor Summary**

`TrainTestEvaluator(TrainTestExperimenter experimenter)`


| **Method Summary** | |
| --- | --- |
| `TrainTestExperimenter` | `getExperimenter()`             public Evaluation[] getEvaluations() { return m\_evaluations; } |
| `uk.ac.ed.inf.utils.database.TableRow` | `trainTestValidate()`             Evaluates a `MultiLabelLearner` via train/test-validation |

**Methods inherited from class uk.ac.ed.inf.enzml.mulan.learn.MulanCrossEvaluator**

`evaluate, getArffFilePath, getEndTime, getEvaluationMeasuresRows, getLearner, getStartTime, getXmlFilePath, internallyCrossValidate, setEndTime, setEvaluationMeasures`

**Methods inherited from class uk.ac.ed.inf.enzml.mulan.learn.EvaluatorNoAUC**

`evaluate`

**Methods inherited from class mulan.evaluation.Evaluator**

`crossValidate, crossValidate, evaluate, setSeed, setStrict`

**Methods inherited from class java.lang.Object**

`equals, getClass, hashCode, notify, notifyAll, toString, wait, wait, wait`

**Constructor Detail**

### TrainTestEvaluator

```
public TrainTestEvaluator(TrainTestExperimenter experimenter)
```


**Method Detail**

### getExperimenter

```
public TrainTestExperimenter getExperimenter()
```

:   **Description copied from class: `MulanCrossEvaluator`**
:   public Evaluation[] getEvaluations() { return m\_evaluations; }

    :   **Overrides:**: `getExperimenter` in class `MulanCrossEvaluator`

---


### trainTestValidate

```
public uk.ac.ed.inf.utils.database.TableRow trainTestValidate()
```

:   Evaluates a `MultiLabelLearner` via train/test-validation


---


|  |  |  |
| --- | --- | --- |
| **Overview** \| **Package** \| **Class** \| **Use** \| **Tree** \| **Deprecated** \| **Index** \| **Help** | |  |
| **PREV CLASS**   **NEXT CLASS** | **FRAMES**    **NO FRAMES**     **All Classes** |  |
| SUMMARY: NESTED \| FIELD \| CONSTR \| METHOD | DETAIL: FIELD \| CONSTR \| METHOD |  |


---
